# Patterns of Pelvic Radiotherapy in Patients with Stage II/III Rectal Cancer

**DOI:** 10.1155/2013/408460

**Published:** 2013-10-02

**Authors:** Timothy L. Fitzgerald, Emmanuel Zervos, Jan H. Wong

**Affiliations:** ^1^Leo Jenkins Cancer Center, 600 Moye Boulevard, Greenville, NC 27834, USA; ^2^Division of Surgical Oncology, East Carolina University Brody School of Medicine, 600 Moye Boulevard Room 4s24 Greenville, NC 27834, USA; ^3^Lineberger Comprehensive Cancer Center, University of North Carolina, 101 Manning Drive Chapel Hill, NC 27514, USA

## Abstract

High-level evidence supports adjuvant radiotherapy for rectal cancer. We examined the influence of sociodemographic factors on patterns of adjuvant radiotherapy for resected Stage II/III rectal cancer. *Methods*. Patients undergoing surgical resection for stage II/III rectal cancer were identified in SEER registry. *Results*. A total of 21,683 patients were identified. Majority of patients were male (58.8%), white (83%), and with stage III (54.9%) and received radiotherapy (66%). On univariate analysis, male gender, stage III, younger age, year of diagnosis, and higher socioeconomic status (SES) were associated with radiotherapy. Radiotherapy was delivered in 84.4% of patients <50; however, only 32.8% of those are >80 years. Logistic regression demonstrated a significant increase in the use of radiotherapy in younger patients who are <50 (OR, 10.3), with stage III (OR, 1.21), males (OR, 1.18), and with higher SES. *Conclusions*. There is a failure to conform to standard adjuvant radiotherapy in one-third of patients, and this is associated with older age, stage II, area-level of socioeconomic deprivation, and female sex.

## 1. Introduction

In contrast to colon cancer, transmural (T3 and T4) and node-positive rectal cancer has a propensity for local recurrence. Based on a significant morbidity of local-regional failure and evidence from prospective randomized trials demonstrating improvement in disease-free survival time and local-regional recurrences [1], the National Cancer Institute sponsored Consensus Conference convened in 1990 and recommended that postoperative chemoradiotherapy should be administered in all patients with stages II and III rectal cancers [2]. Despite these recommendations, adoption of these guidelines into clinical practice has been, at best, uneven [3–6]. 

There is little debate that chemoradiotherapy reduces local-regional failures and improves survival in stages II and III rectal cancer. The Swedish Rectal Cancer Trial randomized patients to preoperative radiotherapy versus surgery alone. The investigators demonstrated improved survival and local control rate in patients receiving a short-course high-dose preoperative radiotherapy [7]. The German Rectal Study Group [8] randomly assigned patients with clinical stages II and III rectal cancer to either preoperative or postoperative chemoradiotherapy, and they provided convincing evidence that preoperative chemoradiotherapy significantly improves the local recurrence rate than postoperative chemoradiotherapy. However, no difference in overall survival rate at 11 years of follow-up was observed despite being adequately powered to address this question [9]. Based on this compelling high level evidence, the National Comprehensive Cancer Network (NCCN) recommends radiotherapy (ideally preoperative) for most patients with stage II/III rectal cancers [10]. 

We have previously demonstrated that significant numbers of patients undergoing curative surgical resection of stage II/III rectal cancer do not receive radiotherapy [3, 6, 11]. It is imperative that patterns of care for patients in whom guideline recommended radiotherapy is not delivered be understood. Omission of treatment in at-risk patients is associated with outcome inequality [12, 13]. This study sought to examine, in a large population-based cancer registry, adherence to National Cancer Institute Consensus guidelines for the use of adjuvant radiotherapy in resected stage II/III rectal cancer. 

## 2. Methods

### 2.1. Data Source

This study utilized data obtained between 1998 and 2007 from the Surveillance Epidemiology and End Results-(SEER-) 17 database. The SEER-17 program is the largest population-based US cancer registry that collects information on incidence, prevalence, and survival rate from specific geographic areas representing 28% of the US population. SEER routinely collects surgical and radiation data but lacks any information on chemotherapy or site of first recurrence.

### 2.2. Patients

We used the SEER-17 database for patients diagnosed with rectal cancers. All patients were restaged using current American Joint Committee on Cancer Criteria. In this study, we included patients with either stage II or stage III rectal cancer, those who had undergone curative surgical resection of the primary tumor (site-specific surgery codes 30, 40, 50, 60, 70, and 80), and those who were documented to have either received or not received pelvic radiation. Patients with SEER historic stage A disease with nonregional metastatic disease at the time of diagnosis (M1), or those who had missing treatment information (i.e., delivery of radiotherapy was not documented) were excluded. The following variables were examined: age, race, gender, year of diagnosis, delivery of radiotherapy, and county-level census data (median family income, percent families below poverty, percent high school education, percent unemployed, and percent white-collar occupation). 

### 2.3. Determination of Socioeconomic Status

We utilized a validated index initially described by Robert et al. [14], which is employed by others to define area-level socioeconomic status (SES) in large databases. Three domains were defined using the following county-level data: education (percent of high school graduates), income (median income and percent below poverty), and employment (percent unemployed and percent white-collar occupation). Each factor was divided into one of 5 quintiles, with one reflecting the lowest socioeconomic quintile. After the quintiles were clearly defined, an overall SES index was derived by adding each factor; these scores were again divided into quintiles to derive a score from 1 to 5, with one reflecting the lowest SES and five the highest SES.

### 2.4. Statistical Analysis

Data was extracted utilizing SEER∗Stat 7.0.5 using the case-listing function and exported to the SAS platform JMP (SAS Institute Inc., Cary, NC, USA) for analysis *χ* ^2^, and Student's *t*-tests were utilized where appropriate. Adjusted analysis was performed with multivariate regression.

## 3. Results

### 3.1. Patient Characteristics

A total of 21,683 patients met the inclusion criteria for this study.  Table 1 summarizes the demographics of the study patient population stratified by whether or not they received radiation. Nearly one-third of patients (32.3%) did not receive adjuvant radiotherapy. A majority of patients had stage III disease (54.9%), were aged >60 years (64.3%), white (83%), and males (58.8%).

Young patients were far more likely to receive radiotherapy than older patients (*P* < 0.0001). Only 33.5% of patients >80 years of age received adjuvant radiotherapy. In contrast, 84.4% of patients <50 years of age received adjuvant therapy. The percentage of patients who received adjuvant radiotherapy decreased with each successive decade of life. Other factors associated with the utilization of adjuvant radiotherapy include patients with stage III disease (stage III, 70.6% versus stage II, 64.3%; *P* < 0.0001) and gender (male, 70.3% versus female, 64.1%; *P* < 0.0001). In addition, we observed that patients residing in lower SES index counties were less likely to receive adjuvant therapy than individuals who resided in higher SES index counties. However, we were unable to define any difference in the delivery of radiotherapy between different racial groups. 

### 3.2. Multivariate Analysis of Sociodemographic Factors

In order to better understand factors associated with delivery of adjuvant radiotherapy, a bivariate logistic regression analysis was performed. Patients greater than 80 years old were significantly less likely to be treated with adjuvant radiotherapy than any other age group (Table 2). Comparing patients ≤50 to those ≥80, the likelihood of adjuvant radiotherapy was 10 times greater (odds ratio 10.3 and *P* value 0.001). The odds of being treated decrease progressively with increased age. Patients with stage III rectal cancer were significantly more likely to be treated with radiotherapy than those with stage II disease (odds ratio 1.21 and *P* value <0.0001). Men were more likely to be treated than women (odds ratio 1.18 and *P* value <0.0001). Socioeconomic status was also linked to use of adjuvant radiotherapy. This association, although statistically significant, was fairly modest. The chance of receiving radiotherapy was approximately 10% greater for patients residing in the highest SES level counties when compared to the lowest. We also noted a trend over time for an increase in use of radiotherapy, odds ratio of 1.41 for treatment in 2007 when compared to 1998, *P* = 0.001.

## 4. Discussion

 The optimal management of a patient with rectal cancer involves a multidisciplinary team whose members include surgeons with expertise in total mesorectal excision (TME), radiation oncologists, medical oncologists, and ancillary support staff. Despite dramatic improvements in local control and survival with the introduction of neoadjuvant and adjuvant radiotherapy regimens, not all patients are treated with radiotherapy [1, 7, 9]. Utilizing a large population-based cancer registry, we report a failure to deliver radiotherapy in one-third of patients with stage II/III rectal cancer. Older age, stage II, area-level socioeconomic deprivation, and female gender were associated with a decreased likelihood of adherence to consensus guidelines.

 We found that there was a linear decrease in the use of radiotherapy with increasing age. Patients less than 50 years old were treated with radiotherapy in 84.4% of cases, whereas only a third of patients greater than 80 years old were treated. This finding was maintained on logistic regression. Patients less than 50 were 10 times more likely to be treated with radiotherapy when compared to those older than 80 (Table 2). Ageism (reflected by undertreatment of older patients) is well documented for colorectal cancer. Such treatment differences are not explained by comorbidities or poor tolerance of treatment regimens [15–17]. Older patients generally derive a similar benefit from adjuvant therapy and can tolerate such regimens [17–20]. Despite this, older patients with colorectal cancer are less likely to be treated with adjuvant therapy [16, 17, 21–24]. Omission of adjuvant radiotherapy in older, medically fit rectal cancer patients may compromise not only local recurrent but survival as well. 

We found that men were more likely to be treated with adjuvant radiotherapy. Men were treated in 70% of cases, whereas women received radiation in only 64% of cases. On logistic regression, this difference persisted, with nearly a 20% increase in treatment for men compared to women. This phenomenon of omission of adjuvant therapy for women with colorectal cancer has been documented in both the United States and The Netherlands [21, 25]. Since there is no reason to expect that women would not derive a similar treatment benefit, the reason of this difference is unclear. 

Lower socioeconomic status is associated with treatment and outcome disparities for patients with colorectal cancer [26, 27]. We found area-level socioeconomic deprivation, as reflected in a validated socioeconomic index, to be associated with decreased use of adjuvant therapy. These findings are similar to other investigators who have noted a slower integration of advances in medical treatment of colorectal cancer for patients residing in counties with lower SES [26]. This slow adaptation of medical innovation has resulted in increasing outcome inequality in regions with area-level SES deprivation.

The lower treatment rates for stage II cancer reported here may, in part, be appropriate. There is some controversy regarding the treatment of T3N0 rectal carcinoma. There may be little benefit to radiotherapy for patients with T3N0 rectal cancer resected using total mesorectal excision with negative radial margins if there is minimal invasion of the mesorectum [28]. These factors are not documented in the SEER registry; as a result, it is impossible to determine whether deviation from standard radiotherapy was rational [29]. 

Although SEER data is not robust enough to make definitive conclusions regarding the effects of radiotherapy on survival, the potential impact of optimal local therapy on overall survival rate should not be overlooked. Educational initiatives and/or centralization designed to improve the quality of care for rectal cancer patients suggest that optimal local therapy can impact not only local recurrence rates but also overall survival rate. The University of Erlangen implemented a strategy to improve the quality of surgery in rectal cancer by routine use of standardized TME surgery. The 5-year local recurrence rate after implementation decreased from 39.4% to 9.1% and survival rate improved from 50% to 71% [30]. Quality initiatives in other studies have found that systematic education regarding implementation of and adherence to optimal surgical and adjuvant therapy guidelines significantly improves survival rates [5, 31–33]. Educational programs in The Netherlands, designed to increase the use of TME and preoperative radiotherapy, were associated with a 9% improvement in 5-year survival rates [33]. Similar results were noted in British Columbia [34]. Jullumstro found that decreased adherence to guidelines in Norwegian patients was associated with an increase in local recurrence and mortality [5]. These findings are not unique to rectal cancer. Women with node-positive breast cancer are generally considered to have systemic disease, but they derive survival benefit from postmastectomy radiation [35, 36]. 

Rectal cancer is no longer a disease that is adequately treated by surgery alone. A large body of evidence now supports the use of preoperative chemoradiotherapy in patients with stage II/III disease along with TME. In this report, utilizing a large population-based database, we demonstrate that pelvic radiotherapy is omitted after presumably curative surgery in one-third of cases. Undertreatment is associated with increasing age, female gender, lower SES, and stage II disease. Although limitations in the SEER registry (i.e., lack of data on chemotherapy and comorbidities data) precluded a definitive survival analysis, it is likely that interventions that improve compliance with treatment guidelines will improve survival in groups at-risk for undertreatment. These data imply that adjuvant radiotherapy is a key quality indicator for this disease. 

## Figures and Tables

**Table 1:** Patient demographics of resected rectal cancer patients (SEER-17; 1998–2007).

Variable	Total	Radiation (%)	No radiation (%)	*P* value
*N*	21,684	14,696 (67.8)	6,988 (32.3)	
Age, years				<0.0001
<50	3,151	2,658 (84.4)	493 (15.7)	
50–59	4,592	3,710 (80.8)	882 (19.2)	
60–69	5,266	3,968 (75.3)	1,298 (24.7)	
70–79	5,450	3,279 (60.2)	2,171 (39.8)	
>80	3,224	1,080 (33.5)	2,144 (66.5)	
Gender				<0.0001
Male	12,756	8,972 (70.3)	3,784 (29.7)	
Female	8,927	5,723 (64.1)	3,204 (35.9)	
Race				0.3902
White	18,007	12,175 (67.6)	5,832 (32.4)	
African-American	1,545	1,048 (67.8)	497 (32.2)	
Others/unknown	2,131	1,446 (69.1)	647 (30.9)	
Stage				<0.0001
II	9,774	6,282 (64.3)	3,492 (35.7)	
III	11,909	3,492 (35.7)	3,496 (29.4)	
SES index				<0.0001
1	4,969	3,286 (33.9)	1,683 (33.9)	
2	4,468	2,953 (66.1)	1,515 (33.9)	
3	4,378	3,093 (70.7)	1,285 (29.4)	
4	3,465	2,344 (67.7)	1,121 (32.4)	
5	4,403	3,019 (68.6)	1,384 (31.43)	
Year of diagnosis				<0.0001
1998	1,240	809 (65.2)	431 (34.8)	
1999	1,180	789 (66.9)	391 (33.1)	
2000	2,387	1,568 (65.7)	819 (34.3)	
2001	2,422	1,541 (63.6)	881 (36.4)	
2002	2,430	1,541 (63.6)	826 (34.0)	
2003	2,375	1,563 (65.8)	812 (34.2)	
2004	2,296	1,565 (65.8)	731 (31.8)	
2005	2,459	1,710 (69.5)	749 (30.5)	
2006	2,370	1,703 (71.9)	667 (28.1)	
2007	2,524	1,843 (73.0)	681 (27.0)	

**Table 2:** Logistic regression of factors associated with radiation delivery in rectal cancer patients undergoing surgery, SEER17 1998–2007.

	OR	CI (95%)	*P* value
Age			
<50	10.3	9.13–11.64	<0.0001
50–59	8.09	7.28–8.98	<0.0001
60–69	5.96	5.41–6.57	<0.0001
70–79	2.99	2.73–3.28	<0.0001
>80	1		
Stage			
II	1		
III	1.21	1.14–1.29	<0.0001
Sex			
Male	1.18	1.11–1.26	<0.0001
Female	1		
SES index			
1	1		
2	0.97	0.89–1.07	0.5653
3	1.25	1.14–1.38	<0.0001
4	1.18	1.06–1.30	0.0015
5	1.11	1.01–1.22	0.0301
Year			
1998	1		
1999	1.08	0.91–1.3	0.3758
2000	1.04	0.89–1.12	0.6421
2001	0.96	0.82–1.12	0.5919
2002	1.07	0.91–1.24	0.4243
2003	1.08	0.92–1.25	0.3579
2004	1.13	0.97–1.32	0.1160
2005	1.20	1.02–1.40	0.023
2006	1.33	1.13–1.56	0.0004
2007	1.41	1.20–1.64	<0.0001
